# Short-Chained Linear Scorpion Peptides: A Pool for Novel Antimicrobials

**DOI:** 10.3390/antibiotics13050422

**Published:** 2024-05-05

**Authors:** Tolis Panayi, Spiridoula Diavoli, Vicky Nicolaidou, Christos Papaneophytou, Christos Petrou, Yiannis Sarigiannis

**Affiliations:** 1 Department of Life Sciences, School of Life and Health Sciences, University of Nicosia, 2417 Nicosia, Cyprus; panayi.t@unic.ac.cy (T.P.); nicolaidou.v@unic.ac.cy (V.N.); papaneophytou.c@unic.ac.cy (C.P.); 2 Department of Health Sciences, School of Life and Health Sciences, University of Nicosia, 2417 Nicosia, Cyprus; diavoli.spiridoula@gmail.com (S.D.); petrou.c@unic.ac.cy (C.P.)

**Keywords:** scorpion venom, venom peptides, AMPs, antimicrobials, therapeutics

## Abstract

Scorpion venom peptides are generally classified into two main groups: the disulfide bridged peptides (DBPs), which usually target membrane-associated ion channels, and the non-disulfide bridged peptides (NDBPs), a smaller group with multifunctional properties. In the past decade, these peptides have gained interest because most of them display functions that include antimicrobial, anticancer, haemolytic, and anti-inflammatory activities. Our current study focuses on the short (9–19 amino acids) antimicrobial linear scorpion peptides. Most of these peptides display a net positive charge of 1 or 2, an isoelectric point at pH 9–10, a broad range of hydrophobicity, and a Grand Average of Hydropathy (GRAVY) Value ranging between −0.05 and 1.7. These features allow these peptides to be attracted toward the negatively charged phospholipid head groups of the lipid membranes of target cells, a force driven by electrostatic interactions. This review outlines the antimicrobial potential of short-chained linear scorpion venom peptides. Additionally, short linear scorpion peptides are in general more attractive for large-scale synthesis from a manufacturing point of view. The structural and functional diversity of these peptides represents a good starting point for the development of new peptide-based therapeutics.

## 1. Introduction

Countless lives have been saved due to antibiotics transforming modern medicine. However, there is a rapid emergence of antibiotic resistance that endangers their efficacy. Antibiotic resistance in pathogenic bacteria has become a major concern in healthcare facilities where nosocomial pathogens are prevalent. The latest data are particularly alarming as the World Health Organization’s (WHO) new Global Antimicrobial Surveillance System (GLASS) shows widespread antibiotic resistance in over 3 million laboratory-confirmed infections reported by 24,803 surveillance sites in 70 countries [1]. The report indicates that the proportion of antimicrobial-resistant bloodstream infections was 36.6% for *Escherichia coli* resistant to third generation cephalosporins and 24.9% for methicillin-resistant *Staphylococcus aureus* (MRSA) [2]. According to the European Centre for Disease Prevention and Control (ECDC), for the antimicrobial groups under regular surveillance (fluoroquinolones, third generation cephalosporins, aminoglycosides, and carbapenems), there were various levels of resistance observed in major nosocomial pathogens. For example, 54% of all *E. coli* isolates were resistant to at least one of these antibiotics, 20.1% for *S. aureus*, 38% for *Klebsiella pneumoniae*, 30.1% for *Pseudomonas aeruginosa,* and 65.6% for *Acinetobacter* spp. [3].

**Figure 1:**
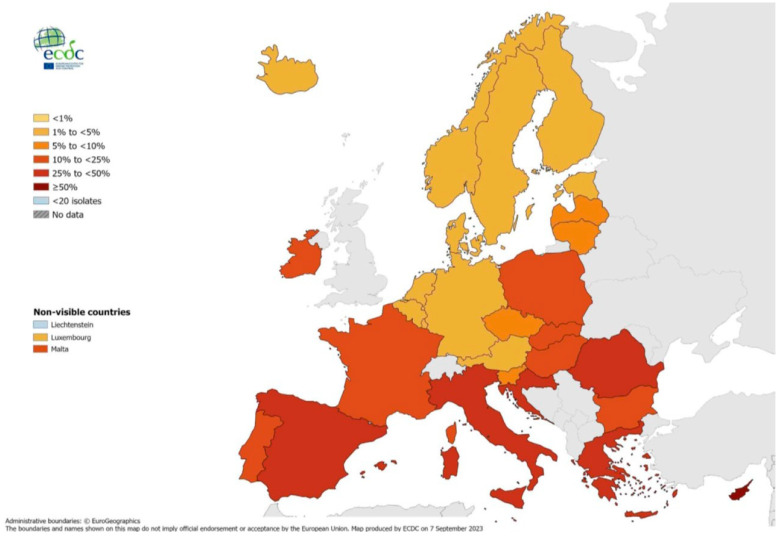
Percentages of *Staphylococcus aureus* invasive isolates resistant to methicillin (MRSA), by country, EU/EEA, 2022 [3]. The figure shows the percentage of *Staphylococcus aureus* invasive isolates resistant to methicillin (MRSA) compared to the total number of *Staphylococcus aureus* isolates across countries in the EU/EEA. Values in southern and south-eastern countries are generally higher than in northern countries. For example, the MRSA isolates accounted for up to 5% in Sweden and up to 50% in Cyprus.

Notably, the antibiotic resistance percentages exhibit significant variations across Europe, with resistance percentages in southern and south-eastern countries being generally higher than in northern countries. For example, in Sweden, invasive *S. aureus* isolates resistant to methicillin (*MRSA*) accounted for up to 5% of all *S. aureus* isolates and up to 50% in Cyprus, as shown in Figure 1 [3]. In order to address this, the WHO has published a list of antibiotic-resistant microorganisms according to the necessity of novel antibiotics against them as shown in Table 1 [4]. However, the prospects for developing new antibiotics have essentially stalled due to economic and regulatory obstacles [5]. The data are rather disturbing: of the 18 largest pharmaceutical companies, 15 have abandoned the antibiotic field, and the merging of others has substantially reduced the number and diversity of in-house research teams. This is because, as far as pharmaceutical companies are concerned, developing antibiotics is not considered a financially savvy investment. Instead, the current focus is on developing more lucrative pharmaceuticals such as chemotherapy drugs [5]. Moreover, academic research has been scaled back due to funding cuts brought on by the economic crisis, especially in southern Europe. Notably, there are currently only 27 new antibiotics in development for the treatment of priority pathogens like carbapenem-resistant *Enterobacteriaceae (CRE), P. aeruginosa, and Acinetobacter baumannii,* down from 31 in 2017. Out of these 27, only 6 meet at least one of the four WHO innovation criteria [6].

In its more recent revision of the “*Guideline on the evaluation of medicinal products indicated for treatment of bacterial infections*”, the European Medicines Agency (EMA) stresses the need for new antibiotics in the face of increased bacterial resistance and suggests amendments to the existing guidelines reflecting upon discussions between regulators in the European Union, United States, and Japan. These include revised recommendations for primary endpoints, primary analysis populations, and non-inferiority margins in trials to support specific infection site-specific indications for use [7].

## 2. The Emergence of Antibiotic Resistance

Antibiotic resistance occurs naturally as a bacterial population is exposed to an antibiotic. As the antibiotic kills all the sensitive bacteria within the population, resistant bacteria, which are naturally present in low numbers, survive, and can multiply and potentially spread their antibiotic resistance attributes to other bacteria through various mechanisms, including conjugation, transduction, and transformation [8,9,10]. Importantly, antibiotic resistance can be facilitated and accelerated because of their misuse and overuse, while the pharmaceutical industry is lacking in the development of new antimicrobial drugs [4]. Types of antibiotic misuse in clinical practice include unjustified prescription for conditions such as the common cold and diarrhoea, under-prescription for conditions such as urinary tract infections and sexually transmitted diseases, under-dosing, and a short duration of treatment [11]. It has been shown that treatment indication, choice of agent, or course of antibiotic therapy is incorrect in 30% to 50% of cases [12]. *Clostridium difficile* infections (CDIs) are almost universally caused because of broad-spectrum antibiotics used to treat other infections. These antibiotics clear the intestinal microbiota of competing bacteria, allowing *C. difficile* to “take over” and colonize the gut [13]. All these factors make the development of new-generation antimicrobial alternatives to antibiotics even more essential than ever before. Antimicrobial peptides (AMPs) have the potential to be an alternative tool in this fight.

## 3. The Abundance of Antimicrobial Peptides in Nature

Ubiquitous in nature, antimicrobial peptides (AMPs) are an ancient and vital component of the rapidly acting innate immunity of a variety of organisms like mammals, insects, bacteria, and plants [14,15]. AMPs are small peptides or proteins (30–60 amino acids) demonstrating broad-spectrum antimicrobial activity against bacteria, fungi, and even cancer cells. AMPs can be isolated from a variety of sources such as marine crustaceans [16], snake venoms [17], frogs [18], human immune cells [19], insects [20], cheese [21], fish [22], and others. AMPs as drugs are recognized for being highly selective, efficacious, and, at the same time, relatively safe and well tolerated. Most of them exhibit antibacterial, antifungal, antiparasitic, and antiviral activity. Consequently, there is an increased interest in AMPs in pharmaceutical research and development (R&D) [23,24,25]. However, even though more than 3000 AMPs have been discovered to date, only 7 of these have been approved by the U.S. Food and Drug Administration (FDA) for use against antibiotic-resistant strains of nosocomial pathogens [24]. Approximately 36 AMPs are currently under investigation, 7 under pre-clinical and 29 under clinical investigation [26]. Nowadays, the development of novel antimicrobials is more essential than ever since increasing resistance rates for last-line-of-defence antibiotics like vancomycin (branched tricyclic glycosylated non-ribosomal peptide) have already been reported. AMPs such as LL-37, thanatin, and melittin are highly effective against Gram-positive (G^+^) and Gram-negative (G^−^) bacteria [27,28,29]. However, applying AMPs as a therapeutic tool is not a straightforward process; many potential pitfalls exist—limited pharmacokinetic profile, short half-life, and bioavailability, or inability to cross the Blood–Brain Barrier. 

Some AMPs have been found to be able to kill both G^+^ and G^−^ bacteria, while others target only one of the two, exhibiting specificity. This is attributed to the structural differences between the two Gram types of bacteria. The “classic” mode of action of AMPs involves their ability to cause cell membrane damage, but some AMPs can also inhibit the synthesis of DNA, RNA, or proteins [30]. Some hypothetical models of membrane-cavity formation, such as barrel-stave, toroidal-pore, carpet, and detergent-like models, have been proposed. In the barrel-stave model, the AMPs’ monomers aggregate and then penetrate the cell membrane, forming channels that result in cell death. AMPs that exhibit this mode of action should have a minimum length of ~22 residues in the case of an α-helix or ~8 residues in the case of a β-sheet to span the lipid bilayer; hence, this model likely does not apply in the case of short-chain linear scorpion peptides. A few AMPs have been shown to follow this model [31]. The toroidal-pore model is also known as the wormhole model. Despite similarities to the barrel-stave model, an important difference is that the peptide helices insert into cell membranes and bind with the lipids, forming toroidal-pore complexes [32]. Several peptides such as magainin II and melittin as well as aurein have been shown to form toroidal pores [33]. In the other two models, carpet and detergent-like models, AMPs act without forming pores in the membrane. In the carpet model, the AMPs accumulate in parallel with the lipid bilayer due to electrostatic and hydrophobic interactions covering the surface of the membrane at a certain peptide concentration, forming a “carpet”. This leads to membrane disruption and micelle formation. The final collapse into micelles is known as the detergent-like model. The carpet model has been proposed for the mechanism of cecropin, LL-37, and indolicin [31]. However, these peptides are too long and out of the range of short-chain peptides. In addition, some AMPs have been found to be effective in killing *Mycobacteria* spp., such as *Mycobacterium tuberculosis*, which fall in neither of the above-mentioned classifications due to the unique structure of the mycobacterial cell envelope [34]. Small peptides like the short-chain linear scorpion peptides can easily cross the membrane without pore formation and spread to the cytoplasm, binding to intracellular targets triggering secondary reactions [35].

Most studies disregard that AMPs could target cell wall components or anchored proteins of the extracellular matrix. A few recent papers elaborating on high-resolution imaging techniques try to capture cellular membrane disruption events and shed light on the mechanism of action of AMPs at the cellular level [36,37]. For the experiments, melittin, alamethicin, and indolicidin have been used. Imaging techniques like cryo-Transition Electron Microscopy (cryo-TEM) and Atomic Force Microscopy (AFM) can be used for the direct visual confirmation of membrane disruption, providing supportive information at the molecular scale.

AMPs can be categorized into different families according to their primary structure, secondary structures, and functions [38]. They can be arranged in three major groups: (i) linear cysteine-free peptides with an α-helical conformation (insect cecropins, magainins, etc.), (ii) cyclic and open-ended cyclic peptides with one to four disulfide bridges (defensins, protegrin, etc.), and (iii) peptides with an over-representation of some amino acids (proline-rich, glycine-rich, histidine-rich, etc.) [39]. The first and second groups include many AMPs derived from scorpion venom. Most of these linear peptides have been identified in venoms in Southeast Asia or Africa. Over 1500 species represent the global distribution of scorpions, and scorpion venoms are complex cocktails of polypeptides. Research has focused mainly on the second category since most lethal toxins acting upon ion channels fall under it [40]. This has left the first category of the linear non-disulfide-bridged peptides derived from scorpions largely unexplored, which is the focus of this review. Short linear scorpion peptides are in general more attractive for large-scale synthesis from a chemical point of view.

## 4. Characteristics and Antimicrobial Activity of Short Linear Peptides

Short linear peptides can be isolated from a whole range of different species of scorpions. These species are spread among the different families that belong to the order *Scorpiones.* The most widespread scorpion family Buthidae occurs across the globe with especially high diversity in northern Africa and the Middle East and includes genera such as *Androctonus*, *Buthus*, *Leiurus*, *Parabuthus*, and *Tityus* [41]. Other families include *Vaejovidae*, found mostly in North America; *Scorpionidae* spread across Africa and the Middle East; *Hormuridae* distributed across the tropic regions of Central America, Africa, and Asia; *Euscorpiidae* spread across the Mediterranean, East and Southeast Asia, and South America; and *Chactidae* found mainly in South America [41].

**Figure 2:**
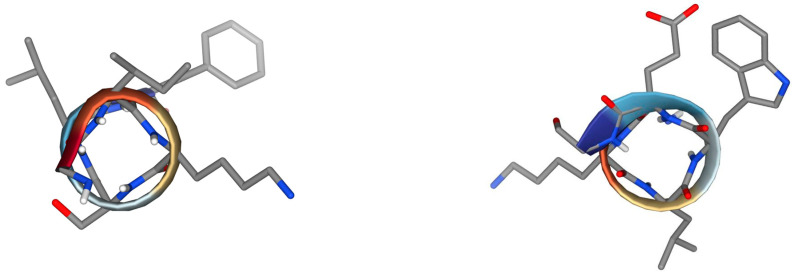
Selected conformations of the peptide fragments GIKSLF (**left**) and GKLWEG (**right**) obtained with PEP-FOLD 4.0 [66]. Hexapeptide GKLWEG is common in peptides pantinin-1 (NDBP-4.20), StCT1 (NDBP-4.8), StCT2 (NDBP-4.15), UyCT1 (NDBP-4.16), and UyCT2 (NDBP-4.17). Hexapeptide GIKSLF is common in the C-terminal region of six peptides, IsCT, IsCT2, UyCT3, HP1090, Um4, and Um2.

The library of the natural, short linear scorpion peptides with 9–19 amino acids from the peptide databases UniProtKB/Swiss-Prot (Group 4—Antimicrobial Peptides), APD3, DRAMP, and ADAM is presented in Table 2. Systematic name NDBP—x.y where x and y stand for the subfamily and peptide number within the subfamily, which should be assigned chronologically, is shown. Several interesting findings, such as common sequences of three to fourteen amino acids, are assigned with different colours. Among the common characteristics of these peptides is the amidation of the C-terminal site, although there is no comprehensive research and clear evidence to prove its necessity. It likely contributes mainly to the stability of the α-helix and possibly the proteolytic stability of the peptides. Interestingly, the peptide pantinin-1 (NDBP-4.20) has a common hexapeptide, **GKLWEG**, with the peptides StCT1 (NDBP-4.8), StCT2 (NDBP-4.15), UyCT1 (NDBP-4.16), and UyCT2 (NDBP-4.17). The hexapeptide **GIKSLF** is common in the C-terminal region of six peptides, IsCT, IsCT2, UyCT3, HP1090, Um4, and Um2. In comparison, the tetrapeptides **SAFK** and **IGGL** are present in five peptides, and the tripeptide **LIP** occurs in seven peptides. These sequences may be important for their antimicrobial activity, their bioavailability, or even their haemolytic activity. Some of the conformations of the common hexapeptides are presented in Figure 2. Through the web application PepDraw, we calculated the net charge of the peptides, their isoelectric point (pI), and their hydrophobicity (www.tulane.edu/~biochem/WW/PepDraw/index.html accessed on 1 March 2024). The computed pI is helpful in choosing a buffer system for the purification and crystallization of a given protein because at this point the peptide or the protein is almost insoluble. Additionally, pI is extremely useful in the purification process in the case of large-scale biotechnological production. Except for StCT1, which displays a pI around 7 (pI = 6.81), the rest of the peptides exhibit pI in the range of 9.74 to 10.59. That means that in neutral pH (~7.4), the peptides are positively charged, developing electrostatic interactions with the negatively charged phospholipid heads of the lipid membranes of the target cells. pI is directly associated with the net charge of a peptide, which is the sum of the charges of the ionizable groups of a peptide. Two peptides, ctriporin and Uy234, exhibit a net charge of +3 while most of the others display +2 except three peptides that present +1, StCT1, Um2, and Cm38. There are no anionic peptides in this group of linear scorpion peptides and in general they are less common.

Hydrophobicity is the free energy associated with transitioning a peptide from an aqueous environment to a hydrophobic environment, like octanol. The scale used to describe this is the Wimley–White scale, an experimentally determined scale, where the hydrophobicity of the peptide is the sum of Wimley–White hydrophobicities [42]. The unit used to express this parameter is kcal per mol, while the pH is assumed to be neutral. According to these parameters, the most hydrophobic peptide is Cm38. Most of these peptides consist of non-polar amino acids presenting increased-to-high hydrophobicity. Hydrophobicity is important for the interaction of the peptides with the membranes. Although increased hydrophobicity without selectivity may result in higher toxicity towards normal cells, there are examples like stigmurin in which the native peptide displays no haemolysis despite its higher hydrophobicity [43].

Moreover, we calculated the Grand Average of Hydropathy Value (GRAVY) for protein sequences using another web application (http://www.gravy-calculator.de/, accessed on 1 March 2024). The GRAVY value is defined by the sum of hydropathy values of all amino acids divided by the protein length. Positive GRAVY values indicate a hydrophobic peptide; negative values indicate a hydrophilic peptide. Most of the peptides present positive GRAVY values (0.51–1.7), indicating the hydrophobic peptide backbones, while the peptides StCT2, UyCT1, UyCT2, and Cm38 exhibit negative values close to 0. Meucin-13, a 13mer isolated from the venom of *Mesobuthus eupeus*, displays the highest GRAVY value. The above calculated properties indicate that these peptides can easily form micelles under physiological conditions, although this property has not been explored. 

Hydrophobicity and hydrophilicity are important parameters for understanding peptide–membrane interactions, as these interactions are responsible for the permeabilization of target cells. In general, highly hydrophobic peptides tend to form pores while the more polar peptides tend to interact in parallel with the negative charges of the membranes. It seems that the ratio of polar to non-polar amino acids guides the mechanism of interactions, influencing the ability of the peptide to cross the membrane [44]. 

Most of the AMPs tend to exhibit strong haemolytic activity interacting with the surface of erythrocytes, usually associated with a higher net charge [45]. There are a few examples in the current literature where when short-chain linear scorpion peptides were modified with lysines to increase the net charge of the native peptide, the haemolytic activity of the newer analogues increased dramatically [46,47]. The haemolytic activity of the short-chain linear scorpion peptides was also computed and predicted in silico by using a web server application(HemoPI: https://webs.iiitd.edu.in/raghava/hemopi/, accessed on 1 March 2024). Toxicity is one of the major hurdles in designing peptide-based therapeutics. In general, certain motifs (e.g., “FKK”, “LKL”, “KKLL”, “KWK”, “VLK”, “CYCR”, “CRR”, “RFC”, “RRR”, and “LKKL”) are more abundant in haemolytic peptides. The algorithm predicts the haemolytic nature of the peptide based on the Support Vector Machine (SVM). Besides the prediction, the platform ranks the peptide analogues based on a simple normalized score derived from the SVM score. Peptides are ranked by probability, between 0 and 1, of being haemolytic, i.e., 1 is very likely to be haemolytic, and 0 is very unlikely to be haemolytic. Most of the peptides exhibit a SVM above 0.5. The highest values are observed for pantinins, ctriporin, and IsCT. Although the net charge seems to be a crucial parameter for the haemotoxic effect of a peptide, there are peptides currently in the market with multiple lysine residues and a net charge of +2 or higher, like lixisenatide administered subcutaneously (under the skin) once a day. The primary structure, the secondary structure, and the net charge contribute to the haemolytic activity. In addition, we used the web application Net Wheels to predict and visualize the helical structures of the peptides [48]. Table 3 displays the Net Wheels projection of the peptides ISCT2, BmKn2, AamAP1, pantinin 2, TSAP-2, UyCT2, and mucroporin. The yellow colour represents the non-polar amino acids, which favour the α-helix conformation, explaining the ability of these peptides to drill the membranes of the bacteria.

**Table 2:** Antimicrobial peptides and their properties calculated with web calculations.

Systematic Name	Name	Amino Acid Sequence	Length	Gravy ^1^	Hydrophobicity (kcal ∗ mol^−1^) ^2^	pI ^2^	Hemo Pi ^3^	Net Charge ^2^	References
NDBP-4.1	IsCT	**ILGKIWEGIKSLF**	13	0.78	10.23	9.74	0.73	2	[49]
NDBP-4.2	ISCT2	**IFGAIWNGIKSLF**	13	1.14	4.69	9.93	0.51	2	[50]
NDBP-4.3	BmKb1	**FLFSLIPSAISGLISAFK**	18	1.54	2.59	9.8	0.54	2	[51]
NDBP-4.4	BmKn2	**FIGAIANLLSKIF**	13	1.67	4.88	9.93	0.6	2	[51]
NDBP-4.5	mucroporin	**LFGLIPSLIGGLVSAFK**	17	1.62	4.59	9.8	0.67	2	[52]
NDBP-4.6	meucin-13	**IFGAIAGLLKNIF**	13	1.7	5.57	9.93	0.52	2	[53]
NDBP-4.7	imcroporin	**FFSLLPSLIGGLVSAIK**	17	1.59	3.9	9.8	0.64	2	[54]
NDBP-4.8	StCT1	**GFWGSLWEGVKSVV**	14	0.51	10.18	**6.81**	0.49	1	[55]
NDBP-4.9	HP1090	**IFKAIWSGIKSLF**	13	1.08	5.95	10.6	0.51	**2**	[56]
NDBP-4.10	ctriporin	**FLWGLIPGAISAVTSLIKK**	19	1.16	6.74	10.6	**0.74**	**3**	[57]
NDBP-4.11	AamAP1	**FLFSLIPHAIGGLISAFK**	18	1.43	5.15	9.8	0.58	2	[40]
NDBP-4.12	AamAP2	**FPFSLIPHAIGGLISAIK**	18	1.23	7.13	9.8	0.48	2	[40]
NDBP-4.13	VmCT1	**FLGALWNVAKSVF**	13	1.21	5.23	9.93	0.51	2	[58]
NDBP-4.14	VmCT2	**FLSTLWNAAKSIF**	13	0.82	4.59	9.93	0.53	2	[59]
NDBP-4.15	StCT2	**GFWGKLWEGVKSAI**	14	0.14	12.82	9.94	0.52	2	[60]
NDBP-4.16	UyCT1	**GFWGKLWEGVKNAI**	14	-0.05	13.21	9.94	0.49	2	[39]
NDBP-4.17	UyCT2	**FWGKLWEGVKNAI**	13	-0.02	12.06	9.94	0.5	2	[39]
NDBP-4.18	UyCT3	**ILSAIWSGIKSLF**	13	1.39	4.07	9.93	0.6	2	[39]
NDBP-4.19	UyCT5	**IWSAIWSGIKGLL**	13	1.14	4.38	10.1	0.59	2	[39]
NDBP-4.20	pantinin-1	**GILGKLWEGFKSIV**	14	0.67	12.04	9.93	**0.82**	2	[61]
NDBP-4.21	pantinin-2	**IFGAIWKGISSLL**	13	1.42	4.76	10.1	**0.83**	2	[61]
NDBP-4.22	pantinin-3	**FLSTIWNGIKSLL**	13	0.94	4.08	10.1	**0.83**	2	[61]
NDBP-4.23	TsAP-1	**FLSLIPSLVGGSISAFK**	17	1.32	5.61	9.8	0.53	2	[47]
NDBP-4.24	TsAP-2	**FLGMIPGLIGGLISAFK**	17	1.55	5.2	9.8	0.52	2	[47]
DRAMP18397	Um4	**FFSALLSGIKSLF**	13	1.49	3.73	9.93	0.51	2	[62]
DRAMP18398	Um2	**ISQSDAILSAIWSGIKSLF**	19	0.83	8.78	6.55	0.51	1	[62]
DRAMP18399	Uy234	**FPFLLSLIPSAISAIKRL**	18	1.33	3.39	11.55	0.55	3	[39]
DRAMP18400	Uy192	**FLSTIWNGIKGLL**	13	0.97	4.77	10.14	0.65	2	[39]
DRAMP18401	Uy17	**ILSAIWSGIKGLL**	13	1.50	5.22	10.14	0.64	2	[39]
DRAMP20775	Ctry2459	**FLGFLKNLF**	9	1.33	3.82	9.93	0.49	2	[56]
DRAMP21024	stigmurin	**FFSLIPSLVGGLISAFK**	17	1.53	3.44	9.80	0.56	2	[63]
DRAMP21251	AaeAP1	**FLFSLIPSVIAGLVSAIRN**	19	1.58	2.78	10.60	0.61	2	[64]
DRAMP21252	AaeAP2	**FLFSLIPSAIAGLVSAIRN**	19	1.46	3.74	10.60	0.66	2	[64]
AP02518	Cm38	**ARDGYIVDEKGCKFACFIN**	19	−0.05	23.50	6.18	0.45	1	[65]

^1^ Gravy calculator: http://www.gravy-calculator.de/. ^2^ PeP Draw: www.tulane.edu/~biochem/WW/PepDraw/index.html. ^3^ HemoPI: https://webs.iiitd.edu.in/raghava/hemopi/, accessed on 1 March 2024.

The peptides IsCT and IsCT2 are both derived from the venom of scorpion *Opisthacanthus madagascariensis* and are analogues with the same C-terminal areas [36]. Screening assays using standard microbiology techniques showed that both peptides were effective against both G^+^ and G^−^ bacteria, the former being much more sensitive to the action of these peptides. When tested in liquid growth assays, it was determined that the peptides caused a steep decrease in microbe growth when a particular concentration threshold was reached. It was proposed that this was due to the peptides facilitating the peptide–lipid interaction, leading to the creation of pores in the phospholipid bilayer membrane [50]. In another study, five synthetic analogues of IsCT with >95% homogeneity were compared between them and to the wild type of the peptide for their antimicrobial activity. While two of the analogues displayed a lower degree of antimicrobial activity, the rest indicated increased activity against both G^+^ and G^−^ organisms [35]. It should be noted that in this study, the wild-type peptide was more effective against the same species of G^−^ microorganisms regarding the MIC when compared to the study of Dai et al. (2002), i.e., *P. aeruginosa* was 2 μM in Lee et al.’s study (2004) and >150 μM in Dai et al.’s study (2002). Among these analogues, [K7, P8, K11]-IsCT was found to have the highest antibacterial activity while presenting no haemolytic activity, which is a desirable outcome given how haemolytic activity is a main drawback in antimicrobial drug development. The authors suggested that the proline-induced bend of P8 is an important determinant of this selectivity [49]. In a study by Tripathi et al., the authors created three different analogues of IsCT (I9K-IsCT, E7K-IsCT, and E7K,I9K-IsCT) by replacing residues at the seventh and ninth positions with lysine residues to investigate both the effects on cytotoxicity and the effects of the introduction of additional positive charges [67]. All three analogues showed significantly reduced toxicity compared to IsCT, with I9K-IsCT and E7K,I9K-IsCT showing a reduction of 99.84% and E7K-IsCT a reduction of 44%. When looking into the antimicrobial activity of the analogues, it was discovered that while IsCT and E7K-IsCT targeted the bacterial membrane, I9K-IsCT and E7K,I9K-IsCT inhibited nucleic acid and protein syntheses in *E. coli* strains without affecting their membrane. While IsCT and E7K-IsCT localised on the bacterial membrane, I9K-IsCT and E7K,I9K-IsCT translocated to the bacterial cytoplasm. Compared to IsCT, analogue E7K,I9K-IsCT showed increased antimicrobial activity against *S. aureus*, *E. coli*, and *P. aeruginosa* strains compared to IsCT (2–6 μM vs. 1–2 μM) [67].

An analysis of the venom of the Australian scorpion *Urodacus yaschenkoi* led to the isolation of four NDBPs: UyCT1, UyCT2, UyCT3, and UyCT5. In their study, Luna-Ramírez and co-workers investigated the membrane interactions and biological activity of these peptides [39]. All the peptides were tested for their antimicrobial activity against multidrug-resistant pathogens including *P. aeruginosa, A. baumannii, S. aureus*, *and E. coli* [39]. This was performed by introducing the peptide in liquid cultures of the tested strains, which included both G^+^ and G^−^ bacteria. The strains were also tested in comparison with ampicillin, which was used as a positive control. The peptide UyCT2 exhibited no activity against G^−^ bacteria at a concentration below 32 μM while the rest of the peptides were effective against both G^+^ and G^−^ strains in comparison to ampicillin. Peptides UyCT1, UyCT3, and UyCT5 were also more effective against G^+^ strains than G^−^ strains with *S. aureus* being the most susceptible strain overall (MIC of 4 μM), while *P. aeruginosa* was the most resistant strain (MIC > 32 μM) [39]. Interestingly, UyCT1 and UyCT2 differ only at the N-terminal site with UyCT1 having an additional Gly, the simplest amino acid, with UyCT1 presenting the strongest activity against the same pathogens but there is no further investigation. These peptides were also tested for their haemolytic activity by incubation with human erythrocytes. All peptides were found to be able to cause 50% erythrocyte lysis (HC_50_) at a concentration range between 14.1 and 100 μM, with UyCT5 displaying the highest haemolytic activity. In a different study, the same research group investigated venom peptides Uy17, Uy192, and Uy234 from the same scorpion as well as peptides Um4 and Um2 from the *Urodacus manicatus* scorpion [62]. These peptides were also tested against both G^+^ and G^−^ bacteria and their haemolytic activity was also determined. Peptides Uy17, Uy192, and Um4 were effective against *S. aureus* at 30μM for Uy17 and 15 μM for the other two. Peptides Uy192 and Um4 were also effective against *E. coli* at 15 μM and 8 μM, respectively. The haemolytic activity (HC_50_) of these peptides ranged between 104.50 and 155.6 μM [62].

Zeng et al. (2013) identified three antimicrobial peptides from the scorpion *Pandinus imperator* using a cDNA library screening: pantinin-1, pantinin-2, and pantinin-3 [61]. The three peptides (13 or 14 residues) exhibit an α-helical conformation and cationic charge and are amphipathic. Protein sequence homology indicates that the amino acid sequence of pantinin-1 shows 64%, 64%, 64%, 57%, and 50% identity to those of the scorpion AMPs IsCT, StCT1, StCT2, UyCT1, and UyCT2, respectively. It was determined that these peptides present strong activity against G^+^ bacteria, whereas their activity against G^−^ bacteria is rather weak. Among the three pantinins, pantinin-1 displayed the strongest activity against *S. aureus* (MIC of 8 μM), while it had relatively weaker action against MRSA (MIC of 14 μM), and even weaker action against other G^+^ bacteria like *Bacillus megaterium* and *Micrococcus luteus* (both MICs were 32 μM). It must be underlined that the sequence of pantinin-1 is completely different from the rest of the pantinins. On the other hand, pantinin-3 exhibited weaker activity than pantinin-1 against *S. aureus* (MIC of 16 μM), but it was more potent against MRSA, *Bacillus megaterium*, and *M. luteus* (MICs of 12 μM, 6 μM, and 8 μM, respectively). The activity of pantinin-2 was weaker in comparison with the two others against most of the strains except for *M. luteus* (MIC of 18 μM). As already mentioned, the activity of all three peptides is much weaker against G^−^ strains, including *E. coli*, *Pseudomonas putida*, and *Salmonella enterica*, with the MIC in some cases being >87 μM. In addition to the bacterial strains, the peptides were also tested against the yeast species *Candida tropicalis* and were all found to be effective at MICs of 16–17 μM. The haemolytic activity of the peptides was found to be very low for pantinin-1 (21% haemolysis at 64 μM) and relatively mild for pantinin-2 and pantinin-3, when considering the MIC against bacteria (100% haemolysis at 64 μM and 32 μM), which was in agreement with the in silico calculations [61].

From the venom of *Buthus martensii Karsch*, Zeng et al. (2004) isolated two highly acidic peptides that did not show homology with other peptides (BmKa1 and BmKa2) and two basic peptides (BmKb1 and BmKn2) [51]. The latter two were tested for their antimicrobial range against both G^+^ (*S. aureus*, *M. luteus*, *Bacillus subtillis*) and G^−^ (*E. coli*, *P. aeruginosa*) bacterial strains. BmKn2 was particularly effective against *S. aureus* and *E. coli* (MICs of 0.6 μg/mL and 1.58 μg/mL) and less effective against *M. luteus*, *B. subtillis*, and *P. aeruginosa* (MICs of 8 μg/mL, 5 μg/mL, and 21.3 μg/mL, respectively). BmKb1 was much less effective against all strains, particularly *M. luteus* and *P. aeruginosa* (MICs of 81.5 μg/mL and 90.8 μg/mL, respectively) [51]. It should also be noted that BmKb2 displayed at least a three-fold lower antimicrobial activity against *P. aeruginosa* compared to the NDBPs IsCT1 and IsCT2 (20% inhibition vs. 70% inhibition) [51].

Mucroporin is the first cationic host defence NDBP isolated from the venom of *Lychas mucronatus* [52]. The authors characterized the peptide via cDNA sequencing and subsequently synthesised it for antimicrobial testing. Using standard microbiology techniques mucroporin (LFGLIPSLIGGLVSAFK) and its optimised analogue, mucroporin-M1 (LFRLIKSLIKRLVSAFK), were tested against several bacterial strains such as *P. aeruginosa*, *S. aureus*, *M. luteus*, and *E. coli.* These included strains resistant to penicillin. Mucroporin was able to inhibit *S. aureus and Bacillus thuringiensis* at 25 μg/mL, *B. subtilis* at 50 μg/mL, and *E. coli and P. aeruginosa* at >100 μg/m, while mucroporin-M1 was more effective at inhibiting *E. coli, B. subtilis*, and *S. aureus* at 12.5 μg/mL, 25 μg/mL, and 5 μg/mL, respectively. Additionally, the replacement with R3 effectively inhibited the penicillin-resistant *S. aureus* compared to the penicillin treatment used as a positive control (10 μg/mL vs. 10,000 μg/mL). Both mucroporins were also able to inhibit liquid cultures of clinical isolates of MRSA at 8 μg/mL [52]. Other studies have shown that both peptides have strong antiviral activity against the Measles virus with an EC_50_ value of 7.15 μg/mL (3.52 μM), SARS CoV with an EC_50_ of 14.46 μg/mL (7.12 μM), and influenza H5N1 with an EC_50_ of 2.10 μg/mL (1.03 μM). The increased activity of mucroporin-M1 was attributed to the enhanced net positive charge of the hydrophilic side group and change in the peptide’s secondary structure [56]. Further research showed that mucroporin-M1 was able to inhibit hepatitis B virus (HBV) replication both in vitro and in vivo by downregulating hepatocyte nuclear factor 4α (HNF4α), which is a nuclear hormone receptor that plays an important role in the replication of HBV. Specifically, the effect of mucroporin-M1 on cells that had been stably transfected with the HBV genome showed that at 50 μM, mucroporin-M1 was able to inhibit the growth of the virus in the transfected cells by 90% and was also shown to be able to reduce the viral production in HBV-infected mice. The putative mechanism behind this is that the peptide activates the mitogen-activated protein kinase (MAPK) pathway, which in turn causes the downregulation of HNF4α [68]. 

Ramírez-Carreto et al. (2012) reported the isolation, identification, synthesis, and characterisation of four peptides (VmCT1, VmCT2, VsCT1, and VsCT2) from the venomous glands of scorpions of the Mexican species *Vaejovis mexicanus smithi* and *Vaejovis subcristatus*. Antimicrobial assays by the broth micro-dilution method showed that while VsCT1 and VsCT2 had no antimicrobial activity against any of the G^+^ and G^−^ strains used, VmCT1 and VmCT2 did. Both VmCT1 and VmCT2 were able to inhibit *S. aureus* at a MIC of 10 μM, *B. subtillis* at a MIC of 20 μM, *P. aeruginosa* at a MIC of 10 μM, and *Streptococcus agalactiae* at a MIC of 10 μM. Both VmCT1 and VmCT2 were also able to inhibit *E. coli* at a MIC of 25 μM and 20 μM, respectively, and *Salmonella typhi* at a MIC of 5 μM and 10 μM, respectively. The peptides were also tested for haemolysis. VmCT1 was only mildly haemolytic, with a maximum of 12% lysis at 50 μM. Meanwhile, the replacement of two amino acids, Gly and Ala, of VmCT1 with Ser and Thr, respectively, made VmCT2 display a strong cytotoxic activity, reaching 84% lysis at 50 μM [59]. Another study by Pedron et al. used helical wheel projection prediction with single and double substitutions to produce seven analogues of VmCT1 and analysed their effect on the antimicrobial and haemolytic activity [58]. The seven analogues and the WT strain were tested against G^−^, G^+^, yeast, and filamentous fungus [44]. Three peptides, [K]3-VmCT1-NH_2_, [K]7-VmCT1-NH_2_, and [K]11-VmCT1-NH_2_ presented higher or equivalent antimicrobial activity when compared to VmCT1 while the rest showed a diminished antimicrobial activity. Also, most of the analogues showed reduced haemolytic activity at the concentration range from 1.56 to 6.25 mM, in which the peptides presented antimicrobial activity against most microorganisms; the percentage of haemolysis was lower than 12% [58].

TsAP-1 and TsAP-2 were isolated from the venom of the Brazilian yellow scorpion, *Tityus serrulatus,* and evaluated for their antimicrobial and anticancer activities. Both peptides showed high degrees of primary structural similarity with the amphibian skin AMPs, phylloseptins and medusins, respectively [47]. Two analogues, TsAP-S1 and TsAP-S2, were produced by the substitution of four non-polar amino acid residues with Lys residues. The MIC and haemolytic activity of the peptides was determined against *S. aureus, E. coli*, and *C. albicans.* TsAP-1 was less effective against all three tested organisms, with MICs ranging between 120μM and 160 μM, whereas TsAP-2 was more effective against the G^+^ bacterium *S. aureus* (MIC of 5 μM) and the yeast *C. albicans* (10 mM). The replacement with Lys in TsAP-S1 improved the activity to 2.5 μM for *S. aureus*/*C. albicans* and to 5 μM for *E. coli*. However, the haemolytic activity increased (4% at 160 mM to 30% at 5 mM, respectively). In TsAP-S2, the replacement had a more pronounced effect, decreasing the MIC for *E. coli* from >320 μM to 5 μM [47].

StCT1 and StCT2 are two peptides isolated at different periods from the venom of *Scorpios tibetanus* that is found in Xizang Autonomous Region, China. StCT1 was synthesised based on cDNA sequencing and tested for its antimicrobial activity against *S. aureus*, *Pseudomonas aeruginosa*, *E. coli*, *Bacillus thuringiensis*, *B. subtilis*, *M. luteus*, and *Enterococcus faecalis* [55]. These included antibiotic-resistant bacterial strains such as methicillin-resistant *S. aureus (MRSA)*, and methicillin-resistant coagulase-negative *Staphylococcus* (MRCNS) [55]. Effectiveness was determined by antimicrobial assays, specifically the microtiter plate method, and recorded as a MIC. Apart from the *S. aureus* indicator strain with a MIC of 12.5 μg/mL, StCT1 had no significant antimicrobial activity against the other organisms tested with its MIC being 50 μg/mL for the penicillin-resistant *S. aureus* and *Enterococcus faecalis* and >100 μg/mL for *Bacillus thuringensis, Bacillus subtilis, Micrococcus luteus, Escherichia coli*, and *Pseudomonas aeruginosa*. It should be noted that the positive control, penicillin G, had a MIC of 6 μg/mL against *S. aureus* and a MIC of >100 μg/mL against clinical strains. StCT1 could completely inhibit the growth of *S. aureus* and *MRSA* in liquid cultures at 12.5 μg/mL and 200 μg/mL, respectively. Later in 2009, Cao et al. discovered, synthesized, and tested StCT2 in vitro and in vivo against a few indicator and clinical strains and its effectiveness was assessed by MIC determination [46]. The peptide was found to be most effective against *S. aureus,* MRSA, PRSA, and other G^+^ strains with a MIC range of 6.25 μg/mL–25 μg/mL, and less effective against G^−^ strains like *E. coli* and *P. aeruginosa* with a MIC range of 50 μg/mL–100 μg/mL, with the latter being the most resistant [60]. The peptide was also tested in an in vivo model where mice infected with MRSA were treated with the peptide and survival was evaluated. At a concentration of 60 mg/kg, the peptide extended the survival over 8 days, similar to a vancomycin positive control, while the untreated animals died after 48 h [60].

Stigmurin was identified in the scorpion *Tityus stigmurus*. Its sequence was determined, synthesised, and tested for its antimicrobial activity by MIC determination [63]. It was tested against *S. aureus* (8.68 μM), *MRSA* (17.37 μM), *E. coli* (>139 μM), and *Candida albicans* (34.75 μM). Stigmurin’s haemolytic activity was 20% at 139.5 μM [63].

From the venom of the North African scorpion *Androctonus amoreuxi,* Almaaytah et al. (2012) identified 18-mer antimicrobial peptides, AamAP1 and AamAP2, which are different at two sites: position two, where Pro replaces Leu, and position 17, where Ile replaces the aromatic amino acid Phe [40]. In the same study, the group determined the antibacterial properties of not only the two peptides but also of a synthetic analogue of AamAP1 named AamAP-S1, where His replaces Lys at position eight, reducing the positive charge of the peptides [34]. The three peptides were tested for their activity against *E. coli, S. aureus*, and *C. albicans.* AamAP1 and AamAP2 presented a MIC of 150 μM and 120 μM against *E. coli,* 20 μM and 48 μM against *S. aureus,* and 64 μM for both against *C. albicans.* The analogue, AamAP-S1, presented significantly more potent activity, decreasing the MIC at 2 μM for *E. coli,* 3 μM *for S. aureus,* and 5 μM for *C. albicans*, whereas the replacement of Lys with His was also advantageous for the haemolytic activity, limiting the haemolysis from 100% for AamAP1 to 53% for the synthetic analogue when red blood cells were incubated with 120μM of the peptides for 120 min [40].

AaeAP1 and AaeAP2 are peptides isolated from the venom of the scorpion *Androctonus aeneas*. Like other venom peptides presented here, both showed significantly increased activity against *S. aureus* (MIC of 16 μg/mL) and *C. albicans* (MIC of 32 μg/mL) compared to the antimicrobial activity against *E. coli* (MIC > 512 μg/mL). One analogue for each peptide, produced by lysine residue substitutions, AaeAP1a and AaeAP2a, were revealed to have increased activity against the three tested organisms: *S. aureus* (MIC of 4 μg/mL), *C. albicans* (MIC of 4 μg/mL), and *E. coli* (MIC of 16 μg/mL) [51]. However, both the WT peptides and the analogues exhibited 100% haemolytic activity (AaeAP1 at 16 μg/mL, AaeAP1a at 32 μg/mL, AaeAP2 and AaeAP2a at 32 μg/mL) [64].

Imcroporin was isolated from the venom of the scorpion *Isometrus maculates* [54]. Its antimicrobial activity was tested against several G^+^ and G^−^ organisms, including antibiotic-resistant strains. As with other similar peptides, imcroporin was more effective against G^+^ strains such as *S. aureus, B. subtilis*, and MRSA, with MICs ranging between 120 μg/mL and 160 μg/mL, and less effective against G^−^ strains like *E. coli* and *P. aeruginosa*, with MICs >100 μg/mL. The peptide also presented haemolytic activity of ~8% at 40 μg/mL, which is acceptable. When used in an in vivo mouse model, the peptide was able to extend 100% survival after 7 days in mice infected with *S. aureus*, while the untreated control had a 100% death after 5 days [54,69].

Ctriporin was isolated from the venom of the *Chaerilus tricostatus* scorpion [57]. Its antimicrobial activity was tested against several G^+^ and G^−^ organisms, which included antibiotic-resistant strains. The MICs of ctriporin against *S. aureus, B. thuringiensis, B. subtilis, M. luteus*, and *C. albicans* range from 5 μg/mL to 20 μg/mL. Meanwhile, its MIC against clinical antibiotic-resistant bacterial strains is 10 μg/mL [43]. Τested in an in vivo mice model infected with *S. aureus*, the peptide significantly reduced the bacterial load and prevented the formation of wounds associated with *S. aureus* infections [57].

Meucin-13 was isolated from the venom of the *Mesobuthus eupeus* scorpion and is an orthologue of other peptides (BmKb1, BmKb2, IsCT, IsCT2) [53]. Like these peptides, meucin-13 has a higher antimicrobial effectiveness against G^+^ bacteria like *B. megaterium* and *M. luteus* (MIC of 0.25–2.9 μM) compared to G^−^ harmful bacteria like *E. coli* and *A. tumerfaciens* (MIC of 6.2–11.8 μM). It also showed antimicrobial activity against fungi and yeasts (MIC of 14.1–42.8 μM). However, the haemolytic activity of the peptide is considered relatively high, 35% haemolytic activity at 6.25 μM [53].

Cm38 was isolated from *Centruroides margaritatus* scorpion venom and purified by two-step chromatography. Its antimicrobial properties were tested using the microdilution assay against *S. aureus* and *K. pneumoniae* [65]. Cm38 was able to completely inhibit the growth of *K. pneumoniae* at 64 μM, while it had minimal effect on the growth of *S. aureus* at the same concentration. When tested for cytotoxic effects against human erythrocytes, no relevant haemolytic activity was observed [65].

The peptide Hp1090 was isolated from the venom of the scorpion *Heterometrus petersi* [56]. Unlike other peptides described herein, this peptide has not been tested for its antimicrobial activity against bacterial strains but rather for its antiviral activity. The peptide was added to the supernatant of cells infected with hepatitis C virus (HCV) at a concentration of 20 μg/mL, which was previously assessed by the MTT assay to have low cytotoxicity (90% survival), using RT-PCR to quantify HCV RNA levels. The results showed that treatment with Hp1090 significantly reduced HCV infection indicated by significantly reduced RNA levels [56]. A more recent study investigated the effects of this peptide, among others, against SARS-CoV-2 spike protein [70]. Similarly, peptide Ctry2459, isolated from the venom of a *Chaerilus tryznai* scorpion, also showed anti-HCV activity, being able to completely inhibit HCV infection in vitro at 20 μg/mL [71]. Ctry2459 was used in the same study to produce two histidine-rich peptides, Ctry2459-H2 and Ctry2459-H3, respectively, which were shown to archive enhanced cellular uptake at 20 μg/mL and also had 1.5-fold and 3-fold reduced haemolytic activity at 203.3 mg/mL and 416.4 mg/mL [71]. It should be noted that this study identified twelve or more venom peptides with anti-HCV activity that were not chosen for further study due to low activity in the initial screening.

## 5. Conclusions

Short-chained linear scorpion peptides with antimicrobial activity represent a novel class of AMPs. Arthropods secrete venom for their protection from bacteria, fungi, and parasites. Short-chained linear scorpion peptides are mainly cytotoxic against G^+^ and G^−^ bacteria while, in some cases, also inhibiting the growth of oral or skin microorganisms like *C. albicans*. Furthermore, most of them present strong haemolytic activity. Several peptides identified to date, such as pantinin-1, BmKbpp, and VmCT1, display low MICs and very low haemolytic activity, confirming their potential as antimicrobials in the pharmaceutical industry. It would be interesting to use modern microscopy techniques like Scanning Electron Microscopy (SEM), Transmission Electron Microscopy (TEM), Fluorescence Microscopy (FM), High-Speed Atomic Force Microscopy (HS-AFM), and cryo-Electron Tomography (cryo-ET) to directly visualize the disruption of the membranes of the bacteria and elucidate the mechanism of action of these peptides.

In general, these peptides have been tested mostly for *MRSA*, *E. coli*, *S. aureus*, and *P. aeruginosa*. Interestingly, despite outbreaks of other microorganisms such as *Streptococcus pneumoniae, Neisseria gonorrhoeae*, etc., which are a high priority for many regions of the world, these peptides have not been tested against them [72,73,74,75]. So, it looks rather rational to test all of them against pathogens classified as high and critical, as well as against their clinical strains responsible for antibiotic resistance cases, hoping to discover novel treatments. Modern peptide drug design could help with the fine tuning and calibrating of the toxicity and specificity of such peptides against selected bacteria, with simple modifications enhancing the possibility of entering and passing clinical phases. Linear peptides of this length can be easily synthesized in a few hours, in a high yield and purity and in a cost-effective way, offering possible solutions in this growing global problem.

It has been poignantly illustrated that in the post-COVID-19 era, it is more essential now than ever that the basic science investigation of novel targets is translated into medical treatments in the case of epidemic, endemic, or pandemic diseases. As illustrated herein, short-chain peptides derived from scorpions hold great promise as novel antimicrobials. As a plethora of scorpion venoms remain unknown or are undergoing investigation, extensive high-throughput screening is needed to focus on the isolation, purification, and characterization of venom components. Artificial intelligence approaches will permit researchers to save time and money and expedite the identification of the most viable candidates for molecular optimization and further drug development.

## Figures and Tables

**Table 1:** WHO priority pathogens list for R&D of new antibiotic [4]. The table shows different species of antibiotic-resistant microorganisms categorized by the WHO according to the necessity of novel antibiotics against them.

**Priority 1: CRITICAL**	
i.	*Acinetobacter baumannii*, carbapenem-resistant
ii.	*Pseudomonas aeruginosa*, carbapenem-resistant
iii.	*Enterobacteriaceae*, carbapenem-resistant, ESBL-producing
**Priority 2: HIGH**	
iv.	*Enterococcus faecium*, vancomycin-resistant
v.	*Staphylococcus aureus*, methicillin-resistant, vancomycin-intermediate and resistant
vi.	*Helicobacter pylori*, clarithromycin-resistant
vii.	*Campylobacter* spp., fluoroquinolone-resistant
viii	*Salmonellae*, fluoroquinolone-resistant
ix.	*Neisseria gonorrhoeae*, cephalosporin-resistant, fluoroquinolone-resistant
**Priority 3: MEDIUM**	
x.	*Streptococcus pneumoniae*, penicillin-non-susceptible
xi.	*Haemophilus influenzae*, ampicillin-resistant
xii.	*Shigella* spp., fluoroquinolone-resistant

**Table 3:** Net wheels projections of selected peptides. Depicted are the Net Wheels projections of the peptides ISCT2, BmKn2, AamAP1, pantinin 2, TSAP-2, UyCT2, and mucroporin. The yellow colour indicates the non-polar amino acids, which favour the α-helix conformation, while red presents the polar basic amino acids, mostly Lys, and green the polar uncharged amino acids, Ser, Thr, or Asn.

ISCT2	BmKn2
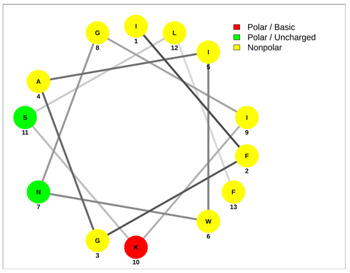	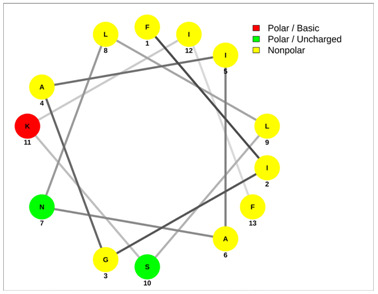
**IFGAIWNGIKSLF**	**FIGAIANLLSKIF**
AamAP1	TSAP-2
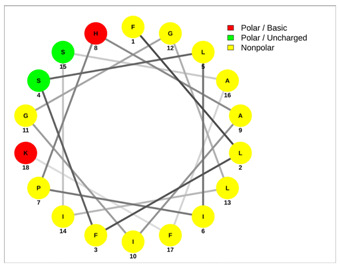	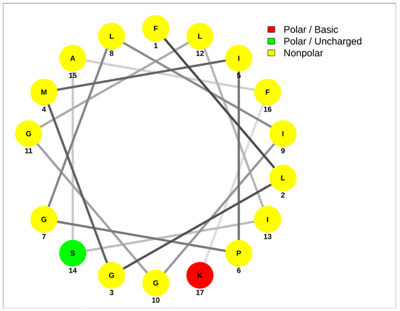
**FLFSLIPHAIGGLISAFK**	**FLGMIPGLIGGLISAFK**
UyCT2	Pantinin-2
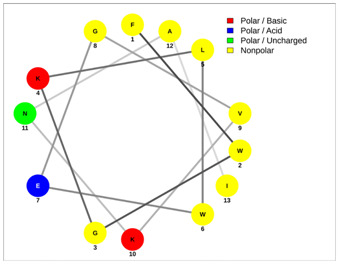	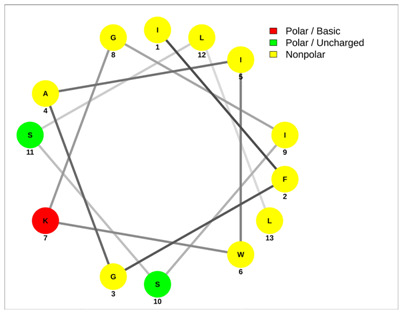
**FWGKLWEGVKNAI**	**IFGAIWKGISSLL**
Mucroporin	Ctriporin
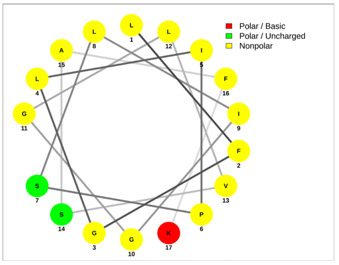	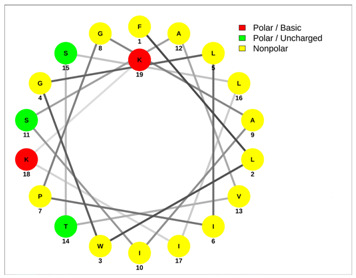
**LFGLIPSLIGGLVSAFK**	**FLWGLIPGAISAVTSLIKK**
